# Mapping Molecular Differences and Extracellular Matrix Gene Expression in Segmental Outflow Pathways of the Human Ocular Trabecular Meshwork

**DOI:** 10.1371/journal.pone.0122483

**Published:** 2015-03-31

**Authors:** Janice A. Vranka, John M. Bradley, Yong-Feng Yang, Kate E. Keller, Ted S. Acott

**Affiliations:** Casey Eye Institute, Oregon Health & Science University, 3181 SW Sam Jackson Park Rd, Portland, Oregon, United States of America; Bascom Palmer Eye Institute, University of Miami School of Medicine, UNITED STATES

## Abstract

Elevated intraocular pressure (IOP) is the primary risk factor for glaucoma, and lowering IOP remains the only effective treatment for glaucoma. The trabecular meshwork (TM) in the anterior chamber of the eye regulates IOP by generating resistance to aqueous humor outflow. Aqueous humor outflow is segmental, but molecular differences between high and low outflow regions of the TM are poorly understood. In this study, flow regions of the TM were characterized using fluorescent tracers and PCR arrays. Anterior segments from human donor eyes were perfused at physiological pressure in an *ex vivo* organ culture system. Fluorescently-labeled microspheres of various sizes were perfused into anterior segments to label flow regions. Actively perfused microspheres were segmentally distributed, whereas microspheres soaked passively into anterior segments uniformly labeled the TM and surrounding tissues with no apparent segmentation. Cell-tracker quantum dots (20 nm) were localized to the outer uveal and corneoscleral TM, whereas larger, modified microspheres (200 nm) localized throughout the TM layers and Schlemm’s canal. Distribution of fluorescent tracers demonstrated a variable labeling pattern on both a macro- and micro-scale. Quantitative PCR arrays allowed identification of a variety of extracellular matrix genes differentially expressed in high and low flow regions of the TM. Several collagen genes (COL16A1, COL4A2, COL6A1 and 2) and MMPs (1, 2, 3) were enriched in high, whereas COL15A1, and MMP16 were enriched in low flow regions. Matrix metalloproteinase activity was similar in high and low regions using a quantitative FRET peptide assay, whereas protein levels in tissues showed modest regional differences. These gene and protein differences across regions of the TM provide further evidence for a molecular basis of segmental flow routes within the aqueous outflow pathway. New insight into the molecular mechanisms of segmental aqueous outflow may aid in the design and delivery of improved treatments for glaucoma patients.

## Introduction

In the human eye, the majority of aqueous humor fluid (approximately 90%) exits the anterior chamber via the conventional outflow pathway, which drains aqueous fluid through the filter-like trabecular meshwork (TM) tissue to Schlemm’s canal.[1] The TM can be divided into 3 separate regions based on location and structure: 1) the outer uveal meshwork, and 2) the deeper corneoscleral meshwork, both regions containing fenestrated beams of lamellae and large, open intertrabecular spaces, and 3) the juxtacanalicular tissue (JCT) that is directly adjacent to the inner wall endothelium of Schlemm’s canal. [2–4] It is composed of JCT cells embedded in a loosely arranged extracellular matrix. Intraocular pressure (IOP) is generated by building resistance to aqueous humor outflow in the TM.[2,5] Aqueous humor outflow resistance is believed to reside within the 7–14 μm of the inner wall of Schlemm’s canal, which is the approximate thickness of the JCT.[2,5–8] The extracellular matrix (ECM) of the JCT is thought to be integrally and extensively involved in generating the outflow resistance, since disrupting it by several methods has been shown to affect outflow resistance.[2,9–11] For example, perfusion of MMPs or their inhibitors resulted in increased or decreased outflow, respectively[9], whereas over-expression of MMP in a steroid-inducible adenovirus increased outflow in perfused anterior segments.[12] In anterior segment organ culture, outflow facility (C) is defined as the flow rate divided by the perfusion pressure, and is inversely proportional to outflow resistance. Glycosaminoglycans (GAGs) and proteoglycans were implicated as integral molecular components of the resistance by perfusion of GAG-degrading enzymes, which increased outflow facility in animals [13–15] Studies using RNAi gene silencing and chemical inhibitors to modify the GAG biosynthesis and structure elicited similar responses on outflow in humans.[3,11,16]

Versican is a large chondroitin sulfate proteoglycan and a component of the ECM of the TM.[17,18] Perfusion of versican RNAi silencing lentivirus or a recombinant HepII domain of fibronectin into human anterior segment organ culture, has been shown to have a direct effect on outflow implicating versican and fibronectin as components of the resistance.[17,19] Additionally, matrix metalloproteinases (MMPs) perfused into anterior segment organ culture increased outflow facility, whereas perfusion of inhibitors of MMPs reduced outflow facility.[9] MMPs -1, -3, -9, and ADAMTS4 have also been shown to be up-regulated, activated, and released in response to increased pressure.[3,10,20] Increase and activation of these enzymes likely target structural ECM proteins of the TM. For example, MMPs -2 and -14 degrade collagen and ADAMTS4 degrades versican, to allow greater aqueous humor outflow. Thus, multiple molecular components of the abundant ECM within the TM are the likely source of aqueous outflow resistance.

Aqueous humor outflow has long been known to be segmental in nature with regions of relatively high and relatively low aqueous outflow around the circumference of the outflow pathway.[21–25] Non-uniform patterns of aqueous humor outflow appear to be universal as they have been demonstrated in mouse, porcine, bovine, monkey and human eyes.[17,26–31] Pigmentation of trabecular meshwork cells was suggested to be a marker for preferential aqueous flow pathways; however, such pigmented regions were not shown to differ in trabecular cell number or ultrastructural characteristics when compared with non-pigmented regions of the aqueous outflow pathway in the same individual.[23,32] Multiple tracers have been used to study the variation in flow including zymosan, latex microspheres, cationic ferritin and fluorescent Qdots. However, cationic ferritin has been used most frequently because of its small size (12nm) and its ability to bind to negatively charged membrane surfaces.[22,24,33–36] In one of the earliest studies done in human eyes, perfusion of cationic ferritin showed the aqueous outflow pathway from glaucomatous eyes to have more segmental variation in the labeling as compared with the outflow pathway from normal human eyes; however, no obvious morphologic changes were associated with the regional changes in cationic ferritin labeling.[34] In support of these findings, another study examined the regional morphological differences in the aqueous outflow pathway and TM around the circumference of the eye and found no significant segmental differences within the aqueous outflow pathway of either normal or glaucomatous individuals in terms of overall ultrastructural distributions.[21] Cationic ferritin perfusion was shown to reduce outflow facility in contrast to perfusion of anionic ferritin, which had no effect on facility, although both showed highly variable distributions around the eye.[22] More recently, studies using dextran beads or fluorescent microspheres perfused into bovine eyes have demonstrated segmental distribution of microspheres at pressures of 15 mmHg and above,[26,27,30,31] and in human eyes at 7 and 30 mmHg.[37] Finally, perfusion of viruses and the pattern of expression of reporter proteins also appear to be segmental.[17,38,39]

In spite of these observations, little is known about how the segmental pattern arises. We hypothesized that the molecular composition in areas of high or low outflow must be different, which either affects or reflects the pattern of outflow. Expression of the proteoglycan versican, was shown to be inversely correlated with outflow regions in human anterior segment organ culture.[17] Additionally, secreted protein acidic and rich in cysteine (SPARC) null mice have recently been shown to have more uniform outflow compared to wild-type littermates suggesting that SPARC may also display segmental differences in expression.[29] Moreover, fibronectin and hyaluronan binding protein were found to have variable patterns of immunostaining, although this was not correlated with tracers.[11,40] The purpose of this study was to further define the nature of segmental fluid flow in the human outflow pathway and correlate patterns of ECM gene expression and proteolytic enzyme activity with high and low flow regions of the TM.

## Materials and Methods

### Anterior segment perfusion culture

Anterior segment perfusion culture is an established technique to study outflow facility *ex vivo*.[10,11,16,17,41] Use of human donor eye tissue was approved by Oregon Health & Science University Institutional Review Board and experiments were conducted in accordance with the tenets of the Declaration of Helsinki for the use of human tissue. Human eye tissue was obtained from cadavers (Lions VisionGift, Portland, OR). Length of time from death to stationary culture was limited 48 hours or less. Anterior segments were placed into serum-free stationary organ culture for 5–7 days to facilitate recovery post-mortem. The age range was 65–97 years and average age of the cadaver eyes for all experiments in this study was 76.29 ± 8.7 years (n = 14). All relevant biological information regarding the donor and known ocular history is listed (S1 Table). Exclusion criteria for donor eyes was: 1) glaucomatous and glaucoma-suspect eyes were not included, and 2) perfused eyes whose flow rates were outside of the range of 1–9 μl/min (or whose facility was outside of the range of 0.125–1.0 μl/min/mm Hg) were excluded from this study. Human anterior segments were perfused with serum-free Dulbecco’s Modified Eagle’s Medium (DMEM) containing 1% Penicillin/Streptomycin/Fungizone, at constant pressure (8.8 mmHg) with an average flow rate of 1–7 μl/min, which is similar to normal physiological rate and pressures (minus episcleral venous pressure) *in vivo*.[11] Data from individual eyes were combined where possible and representative images were used. The number of eyes used for each treatment is noted in the figure legend.

### Tracer Labeling

Anterior segments were perfused continuously, unless otherwise noted, for approximately 5 days. During the final stage of perfusion, fluorescently-labeled microspheres (Invitrogen, Carlsbad, CA) were diluted 1:1000 into PBS, vortexed vigorously, and 200μl of that mixture was injected as a bolus directly in-line into the anterior segment organ culture and perfused for 1 hour. In the case of the soak labeling, the anterior segments were removed from perfusion and allowed to soaking overnight at 37ºC in the fluorescent tracer diluted 1:1000 in PBS. The following day, perfusion was resumed. In the case of the sequential labeling, the first label was perfused in for 1 hour, and then the line and reservoir were washed twice with PBS. The second fluorescent label was then perfused in for 1 hour. The time between sequential labelings was approximately 60 minutes. The labels used in this study are in Table 1.

**Table 1:** Fluorescent microspheres of varying size and modification used in this study.

Label	Color (Ex/Em)	Modification	Size (nm)
Q-Tracker-655	Red (405-615/655)	HIV-Tat	20
Q-Tracker-585	Green (405-545/585)	HIV-Tat	20
Q-Tracker-525	Yellow-Green (505/515)	HIV-Tat	20
Q-dot-655	Red (405-615/655)	ITK amino	20
Fluospheres	Yellow-Green (505/515)	Amine	200
Fluospheres	Red (580/605)	Amine	200
Fluospheres	Red (580/605)	Carboxylate	500
Fluospheres	Yellow-Green (505/515)	Carboxylate	20
Fluospheres	Yellow-Green (505/515)	Carboxylate	200

### Immunofluorescence and Microscopy

At the end of perfusion, TM tissue intended for immunohistochemistry and confocal imaging, was immersion-fixed in 4% paraformaldehyde/PBS for 1 hour at 25ºC. Fluorescent labeling of whole anterior segments were imaged *en face* using a Leica DM500 microscope prior to cutting into 10–12 radial wedges. Frontal sections were then cut with a single-edged razor blade perpendicular to the ocular surface, resulting in a section tangential to the corneoscleral limbus that bisects Schlemm’s canal as described previously.[16,17,27,29] After labeling anterior segments with fluorescent microspheres in organ culture, followed by fixation (as described above), immunostaining was performed as follows. Tissues were incubated in CAS-Block, a universal blocking reagent to saturate the non-specific binding sites (Invitrogen, Grand Island, NY), for 1 hour at room temperature, and then incubated overnight at 4^°^C with one or more of the following antibodies: a versican mouse monoclonal antibody (12C5), a platelet endothelial cell adhesion molecule (PECAM) mouse monoclonal antibody (P2B1), a type VI collagen mouse monoclonal antibody (5C6), and an osteopontin (SPP1) mouse monoclonal antibody (MPIIIB10) all purchased from Developmental Studies Hybridoma Bank, Iowa City, IA, a fibrillin1 mouse monoclonal antibody (MAB1919) purchased from Millipore (Temecula, CA), a SPARC rabbit polyclonal antibody (15274-1-AP) purchased from Proteintech Group (Chicago, IL) or a MMP3 rabbit polyclonal antibody (AAS41420C) from purchased from Antibody Verify (Las Vegas, NV). The PECAM antibody was used as a biomarker of Schlemm’s canal.[42] Primary antibodies were detected with Alexa-fluor 488-conjugated anti-mouse or anti-rabbit secondary antibodies (Invitrogen, Grand Island, NY). Tissue wedges were placed on 0.17 mm Delta T cover glass bottom culture dishes from Bioptechs Inc. (Butler, PA) in Slowfade Gold antifade reagent with DAPI (Invitrogen), and imaged by confocal microscopy using an Olympus FV1000 microscope. Optical sections were acquired using sequential scanning in separate laser channels. Image acquisition settings and number of optical sections in a stack were kept constant when comparing images. Micro-scale analysis of high flow regions was performed by selecting images of high flow regions using Image J software and “Plot Profile” to get graphical output of distance (in μm) versus relative fluorescence intensity. Additionally, confocal images were analyzed using Imaris Bitplane 3-D software (South Windsor, CT) to generate Pearson’s correlation coefficients with selected color channels, as has been described previously.[43] The Pearson’s correlation coefficient is a quantitative statistic relating the degree of overlap between fluorescence signals from two different color channels.[44] Pearson’s coefficient values range from 1.0, indicating complete colocalization, 0, indicating no significant correlation, to -1, indicating complete separation of two signals.[44,45] A Pearson’s correlation coefficient was calculated for each fluorescently-labeled tracer paired with versican immunostaining from raw compressed confocal z-stacks. The Pearson coefficients were averaged and a standard error of the mean was calculated (n = 4).

### Quantitative PCR Arrays

Human anterior segments were mounted for perfusion culture and perfused at constant pressure (8 mmHg) for 48 hours. Amine-modified 200 nm fluorescent microspheres (Invitrogen; 1:1000 dilution) were injected into the perfusion line and perfused into the anterior segments for 60 minutes. Flow was then stopped, and eyes were removed from the organ culture system and viewed *en face* under a fluorescent microscope (Leica DM500, Wetzlar, Germany). Wedges of tissue were cut with a razor blade to separate low and high flow regions of TM. The TM was then dissected from the tissue wedge and RNA was extracted using TRIzol (Life Technologies, Grand Island, NY) according to the manufacturer’s instructions. Total RNA was quantified using a Nanodrop 2000 Spectrophotometer (Thermoscientific, Waltham, MA). Approximately 100 ng total RNA was used as a template for amplification using MessageAmp II aRNA Amplification Kit (Ambion Inc., Life Technologies, Grand Island, NY) according to the manufacturer’s instructions. Equal amounts of amplified RNA (from high and low flow regions of the trabecular meshwork) were reverse transcribed using the RT^2^ First Strand Kit (Qiagen, Hilden, Germany), followed by quantitative PCR with a BioRad/MJ Chromo4 PTC-200 Thermocycler using the RT^2^ Profiler PCR Array, Human Extracellular Matrix and Adhesion Molecules (Catalog # PAHS-013Z, Qiagen). Each 96-well plate includes 84 pathway focused genes, 5 housekeeping genes, a genomic DNA control, 3 wells containing reverse-transcription controls, and 3 wells containing positive PCR controls. Assays for 5 housekeeping genes included in the arrays enable normalization of the data. The genomic DNA control specifically detects nontranscribed genomic DNA contamination with a high level of sensitivity. The reverse-transcription control is an assay that tests the efficiency of the reverse-transcription reaction performed. PCR data was analyzed using the RT^2^ Profiler PCR Array Data Analysis Software, version 3.5 (Qiagen/Sabiosciences), available online at http://www.sabiosciences.com/pcrarraydataanalysis.php. High and low flow regions were selected from 4 different donor eyes and quantitative PCR arrays were performed on each sample. For initial data analysis on the individual PCR arrays, optimal sets of internal control / housekeeping / normalization genes were automatically-selected from each full plate. (The software measures and identifies the genes with the most stable expression via a non-normalized calculation.) The C_t_ values for these genes are then geometrically averaged and used for the ΔΔC_t_ calculations, which represent normalized gene expression values for all genes. Fold change values for each experiment were calculated as relative gene levels in high flow regions in comparison with gene levels in low flow regions using the same data analysis software. Fold-changes were averaged and a standard error of the mean was calculated for all genes in the PCR array.

### Statistical Analyses

The threshold fold-change differences that were semi-arbitrarily determined to be biologically significant were fold-change values that were greater than 1.5 (i.e., genes enriched in high flow regions) and less than or equal to 0.5 (i.e., genes enriched in low flow regions.) Initially, all fold-change values were subjected to an unpaired t-test in comparison with a set of genes that had no change across all experiments (fold-change = 1.0). P values <0.05 were determined to be significant. Data were then assessed using Bonferroni to correct for multiple comparisons. Subsequently, we used the more rigorous SAM (significance analysis of microarrays),[46] version 4.01, with 4 biological replicates, to determine statistically significant gene changes for HF and LF regions. We normalized the raw data using the delta delta Ct values for HF and LF regions. The analysis was two class paired, both with arrays median centered and without centering, 500 permutations. 10 nearest neighbors were calculated for K-Nearest Neighbor imputer. Different Delta Values (for adjusting the false discovery rate) were set to be between 0.264 and 0.61 with similar results. SAM was obtained from (http://www-stat.Stanford.EDU/~tibs/SAM). REXCEL 2013, a high-level interface between R and Excel, was obtained from (http://rcom.univie.ac.at/); R is a free software environment for statistical computing and graphics and more can be found at (http://www.r-project.org/). The data obtained with SAM is shown as fold change gene expression, and is in good agreement with the initial fold change values determined as statistically significant by unpaired t-test and Bonferroni.

### MMP activity assays

Fluorogenic peptide assays were used to quantitate MMP (Sensolyte 520 generic MMP assay kit, Anaspec, Fremont, CA) activity in low and high flow areas of human tissue perfused with Qtracker 594, as described for human anterior segments above. Human tissue was acquired and anterior segments were perfused in organ culture with fluorescent microspheres. Tissues wedges were separated into areas of high and low flow based on fluorescence intensity and the TMs were dissected. Tissue samples were homogenized in the lysis buffer supplied with the kit. Samples were activated by incubation with APMA (*amino-phenyl mercuric acetate*) for 90 minutes and then incubated with the quenched fluorescence resonance energy transfer (FRET) peptide. The MMP peptide is a substrate for MMPs -1, -2, -3, -7, -8, -9, -10, -12, -13 and -14. Upon MMP cleavage, the peptide generates fluorescence, which was measured using a plate reader (Ex/Em = 490/520 nm). To account for the different sizes of tissue wedge, relative fluorescent units (RFUs) were normalized to total protein concentration (μg/ml) in each sample as measured by the BCA assay (Thermo Fisher Scientific, Rockford, IL). Data from multiple regions (n = 5) were averaged and a standard error of the mean was calculated. Significance was determined using ANOVA, where p < 0.05 was considered significant.

## Results

### Segmental Flow in Perfused Human Eyes

**Fig 1:**
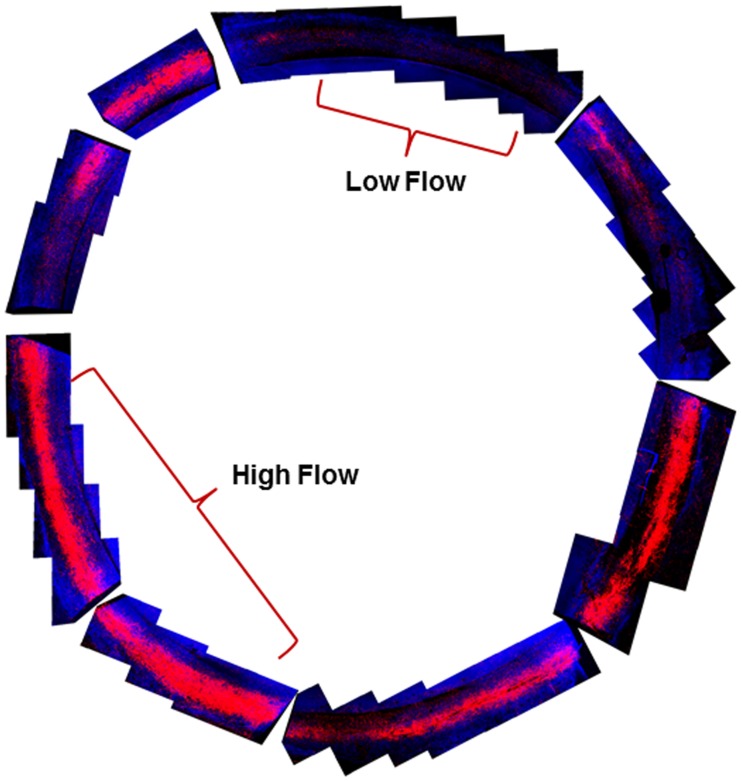
Segmental flow in perfused human eyes. *En face* image of a whole human eye labeled with Qtracker-655 (red) during perfusion. The perfused tissue was cut into approximately 8 wedges and all were imaged *en face* by confocal microscopy. En face images were positionally labeled and then photo-merged digitally to create a collage using Adobe Photoshop software. Regions of high and low labeling demonstrate the segmental nature of outflow with labeled brackets indicating examples of each region. Gaps reflect razor blade cut sites. Blue is autofluorescence of the TM tissue.

Segmental flow was visualized circumferentially within the aqueous outflow pathway in a human donor eye after perfusion of the anterior segment in organ culture with Qtracker-655 quantum dots (HIV-TAT labeled) (Fig 1). Examples of regional differences are indicated by brackets. Regions of high and low fluorescence labeling were distinguishable from each other *en face* and correlate with areas of high and low outflow.

We hypothesized that fluorescent microspheres of varying size and different modifications could show differences in their distribution patterns across the various layers of the TM (for example, in the corneoscleral meshwork versus the JCT region), possibly indicating potential flow routes through this tissue. To evaluate this possibility, a variety of fluorescent tracers (Table 1) were perfused into anterior segment organ culture prior to fixation. Frontal sections of tissues were imaged using confocal microscopy to compare their distributions across the various layers of the TM within high and low flow regions (S1 Fig). Qtracker-655 quantum dots (20nm in size) are predominantly localized within the outer uveal TM beams and corneoscleral meshwork in both high and low flow (regions although at dramatically higher levels in high flow regions (S1 Fig). These Qdots are modified with a HIV-TAT peptide to facilitate cellular uptake and they appear to be taken up most effectively by the uveal TM beam cells. In comparison, the 200nm amine-modified fluorescent microspheres localized throughout the various layers of the TM and into Schlemm’s canal in high flow regions (S1 Fig). The larger tracers seemed to have a wider distribution throughout the TM, particularly in the high flow regions, whereas the smaller tracers appear to be taken up more aggressively by the outer uveal TM cells. Segmental flow appears to be independent of tracer size and modification, but localization of tracers across the various anatomical layers of the TM is not.

Next, we compared distribution of fluorescent tracer which had been perfused in to the anterior segment with fluorescent tracer which had been passively soaked into the anterior segment tissue overnight. Amine- and carboxy-modified fluorescent microspheres were either sequentially perfused into anterior segment organ culture as a control, or the green amine-modified microspheres were passively soaked into the tissues overnight, followed by active perfusion of red amine-modified microspheres. After paraformaldehyde fixation, tissues were cut and frontal images were acquired by confocal microscopy. When both amine- and carboxy-labeled microspheres were sequentially perfused into the tissue they localized to similar areas of the TM (S2 Fig). However, when green fluorescent microspheres were allowed to passively soak into the tissue prior to perfusion of red fluorescent microspheres (S2 Fig), the soaked tracer localized throughout the TM including the low flow regions and into surrounding tissues, whereas localization of the perfused tracer was limited to the putative high flow regions of the TM. This experiment provides further evidence that the perfusion-labeled regions reflect actual fluid flow regions and that segmental difference are not due to the molecular structural components of the TM.

### Mapping the Outflow Regions

**Fig 2:**
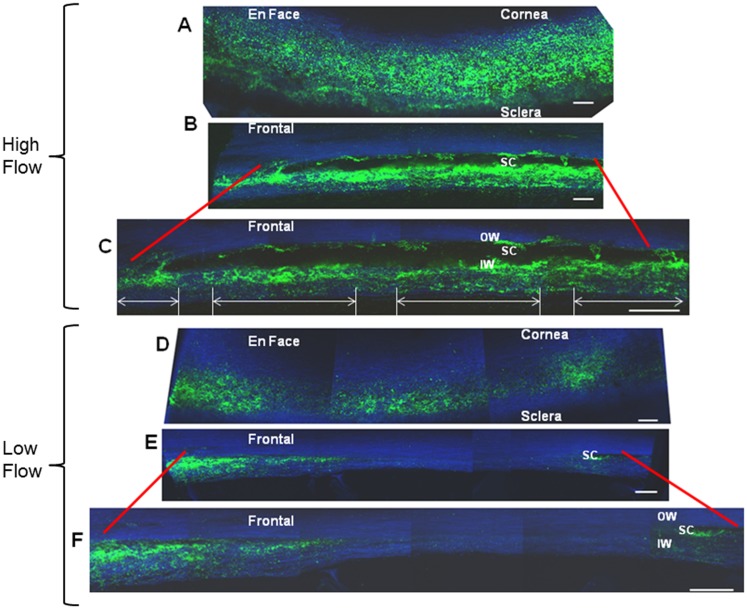
Mapping the flow regions in high and low flow areas of the TM. Human TM sections from high flow (A-C), and medium—low (D-F) flow regions labeled with 200 nm fluorescent amine-modified microspheres (green) are shown *en face* (A and D) or as frontal sections (B, C, E, F). Confocal images of frontal sections show labeling at increasing levels of magnification. High flow regions are shown to contain micro sub-regions of high and low flow (arrows) when viewed at higher magnification (C). Blue color is due to autofluorescence of the TM. Scale bars = 100 μm. OW = Outer wall, SC = Schlemm’s canal, IW = Inner wall.

**Fig 3:**
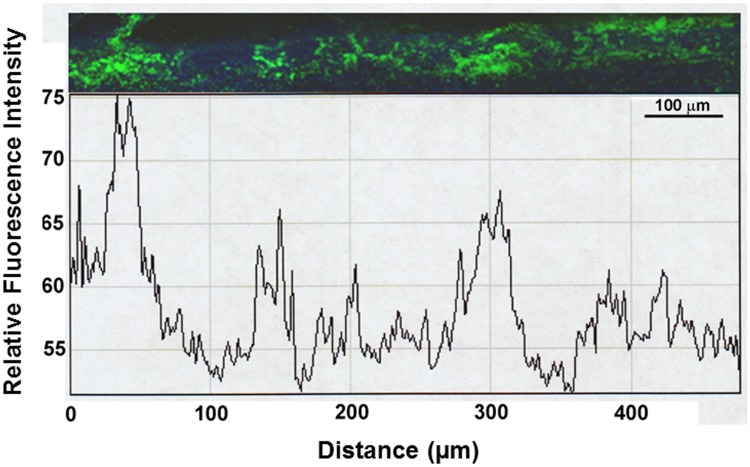
Micro-scale distribution of fluorescent microspheres in a high flow region. High flow regions as shown in Fig 2 were imaged at higher magnification. Relative fluorescent intensity (RFU) was measured across 500 μm and plotted using Image J software. A periodic pattern of micro-flow regions alternating high and low regions (arrows) at approximately every 50–100 μm is shown and originated from a macro high flow TM region. Scale bar = 100 μm.

Next, we further characterized the high and low flow regions. Human anterior segments were perfused with 200 nm green amine-modified fluorescent microspheres and TM tissue was fixed and imaged by confocal microscopy. Representative areas of high and low labeling are shown both *en face* (Fig 2A and 2D) and in frontal sections (Fig 2B-2C and 2E-2F). The representative frontal sections of high flow (Fig 2A-2C) and low flow (Fig 2D-2F) areas are shown at two different magnifications. The low flow regions contained smaller amounts of fluorescent labeling than high flow regions. These images show that at increasing magnification, the distribution of the fluorescent microspheres in high flow regions consists of smaller sub-regions of alternating high flow regions (arrows) interspersed with smaller relatively-low flow regions. These high flow sub-regions were approximately 50–100 μm in length (Figs 2C and 3). Relative fluorescence intensity was plotted across 500 μm of a representative high flow region of the TM to graphically demonstrate the periodic distribution of high and low sub-regions contained within a larger high flow region (Fig 3).

### Perfusion of Labeled Microspheres Selectively Targets the TM

**Fig 4:**
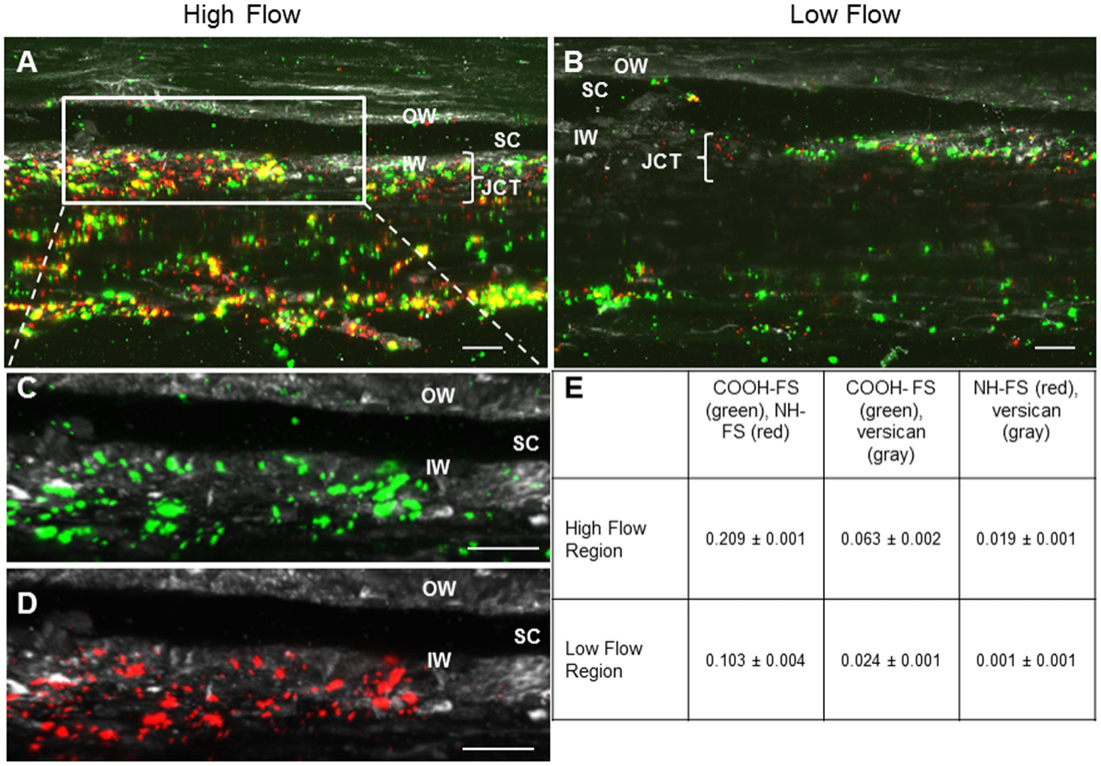
Amine- and carboxy-modified fluorescent microspheres (200 nm) colocalize in high and low flow regions of the TM. Anterior segments were sequentially perfused with 200 nm amine-modified (red) and carboxy-modified (green) fluorescent microspheres prior to fixation and confocal imaging. Confocal images show overlapping red and green in both high (A, C, D) and low (B) flow regions. Immunostaining for versican (gray) localizes to the inner wall and outer wall of Schlemm’s canal and to the JCT region of the TM (A-D). Boxed area in A was zoomed and red and green channels were separated to visualize overlap with versican (gray) (C, D). Pearson’s correlation coefficients (Pcc) were measured using Imaris software in order to determine the amount of colocalization between two signals €. The amine- and carboxy-modified fluospheres colocalize in both high and low flow regions of the TM; n = 3 for each pairing. Scale bars = 20 μm. OW = Outer wall, SC = Schlemm’s canal, IW = Inner wall, JCT = Juxtacanalicular TM.

Versican has been shown previously to selectively label the JCT region of the TM and contains negatively charged glycosaminoglycan side chains which might influence the distribution of amine-modified fluorescent microspheres within the TM. Therefore, fixed tissues that had been perfused with 200 nm amine- and carboxy-modified fluorescent microspheres were immunostained for versican, and high and low flow regions were imaged by confocal microscopy (Fig 4). Distribution of versican immunostain and amine- versus carboxy-labeled microspheres was compared and Imaris software was used to determine Pearson’s correlation coefficients. This provides a quantitative assessment of the colocalization between two signals acquired in separate channels. Surprisingly, amine- and carboxy-modified spheres appeared to colocalize moderately with each other, but had very weak colocalization with the versican immunostaining (Fig 4C-4E).

**Fig 5:**
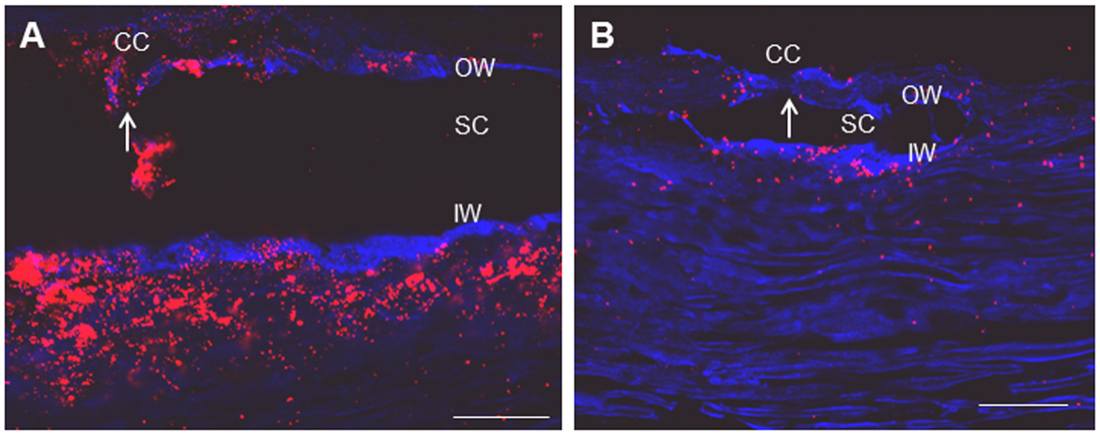
Collector channels are present in high and low flow areas of the TM. Representative images are shown of high (A) and low (B) flow regions of the TM after perfusion with 200 nm amine-modified fluorescent microspheres (red). Fluorescent microspheres appear to accumulate in areas near collector channels (arrows) in both regions. Scale Bar = 50 μm. OW = Outer wall, SC = Schlemm’s canal, IW = Inner wall. Blue is PECAM immunostaining to aid in the visualization of Schlemm’s canal and collector channels, particularly in low flow regions.

It has previously been shown that collector channels are often found in high flow regions, but low flow regions were not studied in detail.[26,37,47] We wanted to determine the likelihood of finding collector channels in low flow regions of the TM. Thus, after perfusion with amine-modified fluospheres, collector channels were imaged using confocal microscopy. Schlemm’s canal was visualized with PECAM immunostain (blue) which specifically localizes to inner wall endothelial cells.[42] Representative images show that collector channels are present both in high flow (Fig 5A) and low flow (Fig 5B) areas of the TM. In both regions, the fluorescent microspheres tended to accumulate in and around Schlemm’s canal adjacent to collector channels when collector channels were present; however no quantitation was done on the relationship between collector channels and bead location.

### Gene Expression Differences in High and Low Flow Regions of the TM

**Fig 6:**
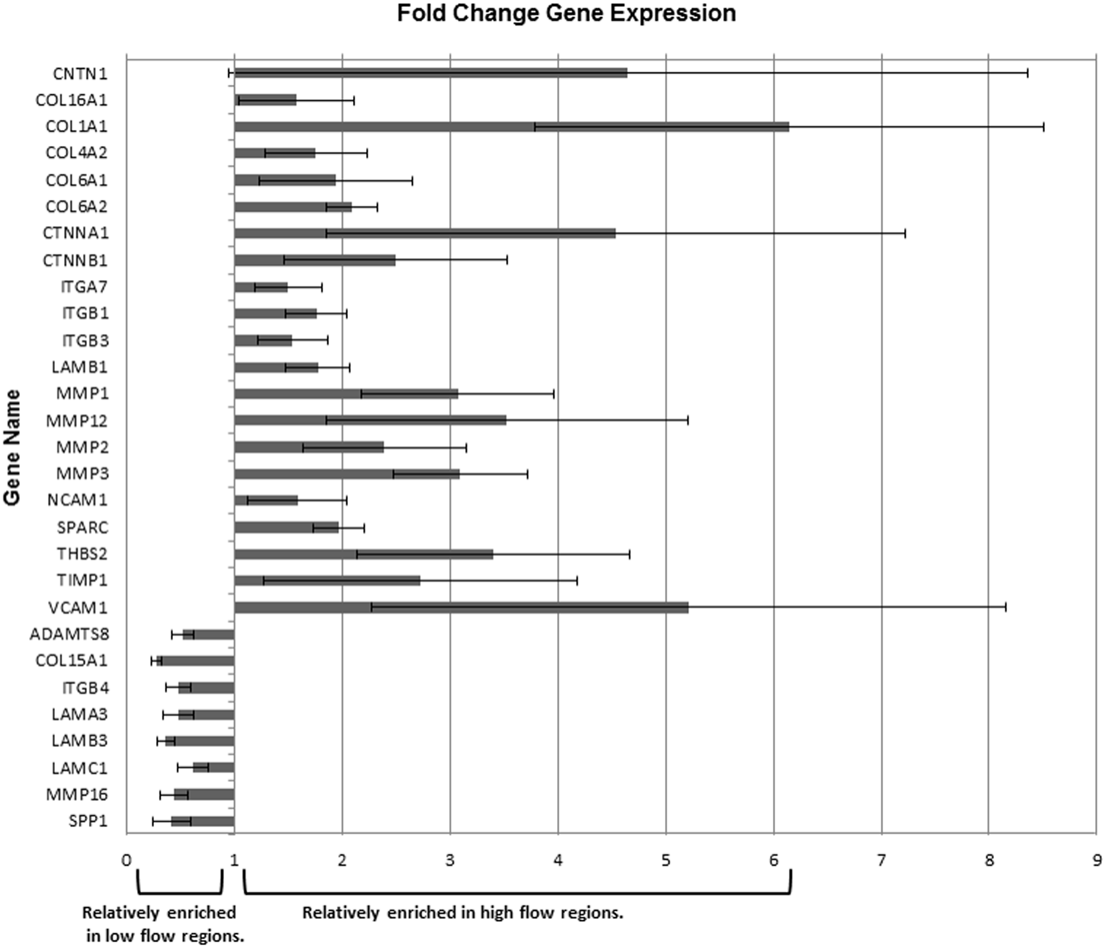
Quantitative PCR array of TM from high flow regions in comparison with low flow regions. Human TM’s were dissected from perfused anterior segments, RNA was isolated, reverse transcribed, and measured using the human extracellular matrix and adhesion molecule quantitative PCR array. Fold change gene expression is shown as either enriched in high flow regions (values greater than 1.0) or enriched in low flow regions (values less than 1.0). All fold changes greater than 1.5 fold and less than 0.5 were considered to be biologically significant. SAM (significance analysis of microarrays), version 4.01, with 4 biological replicates, was used to determine statistically significant fold gene expression changes for HF regions in comparison with LF regions. The raw data using the delta delta Ct values for HF and LF regions was normalized, then subjected to SAM analysis. All fold change genes shown here were determined to be statistically significant by SAM, and biologically significant, by our 1.5 and 0.5 fold criteria. Error bars are the s.e.m.; n = 4 high flow and low flow regions from 4 individual donor eyes.

ECM is thought to be the source of outflow resistance so we hypothesized that ECM gene expression may be different between regions, which may provide clues as to genes that are important to facilitate aqueous humor outflow. In order to determine molecular differences in ECM genes in high and low flow regions of the TM, RNA was extracted and ECM and adhesion gene quantitative PCR arrays were performed (Fig 6). Fold change values are represented graphically and show gene changes in high flow regions in comparison with low flow regions. The collagen genes COL1A1, COL4A2, COL6A1, COL6A2, and COL16A1, were all more enriched in high flow regions, whereas COL15A1 was enriched in low flow regions. The matrix metalloproteinase (MMP) genes MMP1, 2, 3 and 12 were enriched in high flow regions. Conversely, MMP16 was relatively enriched in the low flow regions. Additional differences were seen with the matricellular gene SPARC which was enriched in high flow regions, whereas osteopontin (SPP1) was enriched in low flow regions. Other gene families such as the integrins (ITG) and laminins (LAM) also showed variable expression with some members enriched in high flow regions and others in low flow regions. Thus, there are distinct molecular differences in ECM gene expression levels in high and low flow regions of the TM.

**Fig 7:**
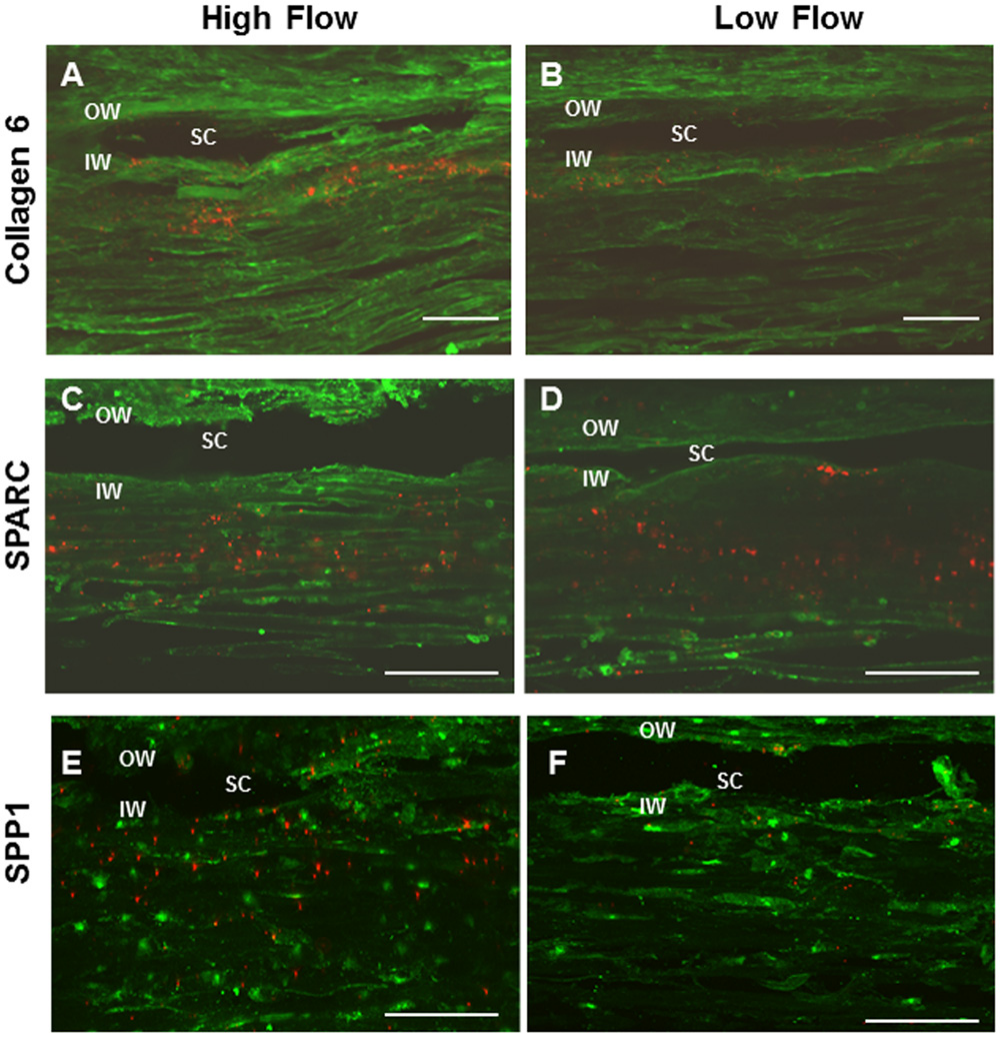
Immunostaining for select ECM proteins in high and low flow regions of the TM. Tissues were perfused with 200 nm amine-modified fluorescent microspheres (red) to separate high (A, C, E) and low (B, D, F) flow regions. Frontal sections were immunostained (green) with antibodies to Type VI collagen (A, B), SPARC (C, D) or SPP1 (E, F) and imaged by confocal microscopy. Scale bar = 50 μm. OW = Outer wall, SC = Schlemm’s canal, IW = Inner wall.

Immunostaining of TM tissues of a select set of proteins, whose genes showed differential expression in high and low flow regions of the TM, was performed to corroborate some of the PCR array data (Fig 7A-7F). Type VI collagen (Fig 7A-7B) and SPARC (Fig 7C-7D) genes were both enriched in high flow regions of the TM and their immunostaining patterns indicate that their proteins are also more enriched in high flow regions, respectively. SPP1, a gene that was slightly more enriched in low flow regions of the TM, only showed minimal differential immunostaining in high (Fig 7E) and low (Fig 7F) flow regions.

### MMP Activity Assay

**Fig 8:**
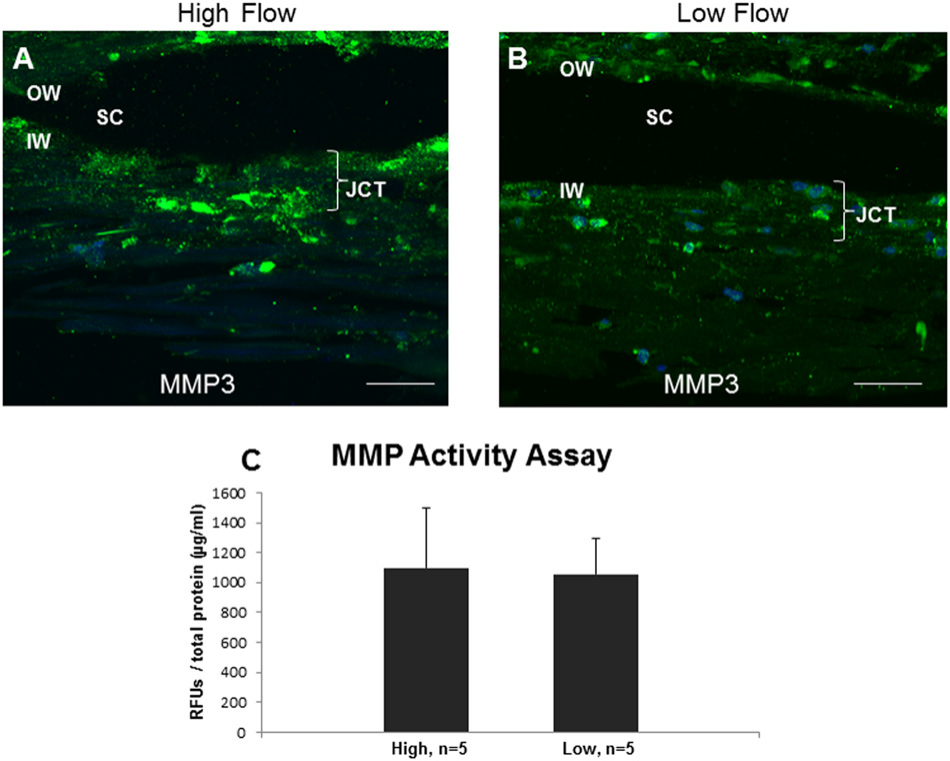
MMP3 immunostaining and MMP activity in high and low flow regions of human TM. Frontal sections of high (A) and low (B) flow regions of human TM from perfused anterior segment organ culture were immunostained with an antibody to MMP3 (green). DAPI is the nuclear stain (blue). Scale bar = 30 μm. OW = Outer wall, SC = Schlemm’s canal, IW = Inner wall, JCT = Juxtacanalicular TM. MMP activity in high and low flow regions of human eyes (C). Results are shown as relative fluorescent units (RFUs) normalized to total protein (μg/ml) in each sample. “N” for each region is listed in the graphs.

The gene expression data shows enrichment of MMP1, 2 and 3 in high flow regions and, conversely, enrichment of MMP-16 in low flow areas. Immunostaining of high and low flow regions of the TM was performed using an antibody to MMP3 (Fig 8A-8B) and showed increased staining in the high flow regions. Regulation of MMP genes does not necessarily equate to increased activity of the enzymes. To quantitate proteolytic enzyme activity in each outflow region of the tissue, an enzyme assay was performed using a peptide that can be cleaved by multiple MMPs (Fig 8C). There was no significant difference in the total proteolytic MMP enzyme activity in the high flow regions of the tissue compared to the low flow regions. This indicates that although there are individual MMP gene expression differences in the high and low flow regions of the TM, these may not be reflected in overall protein levels or enzyme activities measured on the same regions.

## Discussion

In this study, various fluorescent tracers were perfused into human anterior segment organ culture to further define the nature of segmental aqueous humor outflow. Although segmental outflow has been observed for many years, the contributing factors, and its consequences on outflow resistance, remain poorly understood. It is generally believed that regions of high tracer labeling are considered areas of high fluid flow across the TM and into Schlemm’s canal, whereas regions of low tracer labeling are areas of little to no fluid flow. Our studies using actively perfused microspheres versus passively soaked microspheres supports that tracers are labeling outflow pathways. Our analyses of many perfused human eyes showed some variability in the relative amount of high and low flow regions. However, in general, approximately one-third of the each eye was high flow, one-third low flow, and the remainder was medium flow. This is in agreement with a recent study that determined only about one-third of the TM is tracer-labeled and therefore involved in active filtration at any given time.[37]

Initially, we varied the size and the modification of the tracer perfused into the tissue to determine differences in localization patterns throughout the different anatomical layers of the TM. Previous studies have shown that perfused cationic ferritin localizes to different areas of the TM than anionic ferritin and reduces outflow facility.[22] In particular, cationic ferritin was consistently seen on the luminal surface of the inner wall of Schlemm’s canal and variably labeled the JCT and trabecular beam surfaces, whereas anionic ferritin was more prominent in the JCT and intertrabecular spaces.[22] It is unclear whether there might be charge-specific interactions with ECM components in the outflow pathway, or whether net charge patterns within the outflow pathway impact flow marker binding, both of which would affect fluid flow across the TM. Fluorescent microspheres with an HIV-TAT modification (20 nm) localized to the outer uveal TM beam region presumably because of highly-active phagocytosis. Similarly-sized microspheres without the HIV-TAT modification did not accumulate in the outer uveal meshwork, but they were more sparsely distributed throughout the layers of the TM. Larger fluorescent microspheres with amine-modification were more evenly distributed across the TM and, in particular, within the JCT region of the TM

The conventional outflow pathway is an attractive therapeutic target for glaucoma patients since lowering IOP remains the only effective strategy to stop the progression of glaucoma vision loss. However, no drugs are currently in use that target this pathway. In order to target glaucoma drugs to the TM, in-depth knowledge of TM cell biology is required. Gene expression differences between TM beam cells, JCT cells and Schlemm’s canal inner wall cells have been reported.[48–50] It is attractive to speculate that by simply varying the size of the tracer, and optimizing the dose and treatment time, different populations of TM cells might be targeted. This feature would be especially beneficial for targeting drug therapies to distinct TM cell populations.

We compared the active perfusion of fluorescent tracer with the passive soaking of fluorescent tracer to determine whether the patterns of segmental outflow are dependent on perfusion. When fluorescent tracer was allowed to soak into the tissues overnight in the absence of perfusion, it was found in all areas of the tissue and no segmental effect was observed. The same tissues were then perfused with a differently-colored fluosphere, and that tracer preferentially localized within the TM and Schlemm’s canal. This suggests that although all areas can be labeled equally, segmental outflow requires active perfusion and that only the filtration-active areas are labeled. The biological function of the low flow areas remains unclear at this time. However, even though low flow areas are not involved in filtration, the cells in these regions remain viable as HIV-TAT nanoparticle uptake shows. Interestingly, within areas considered to be high flow, imaging at higher magnification showed micro-scale regions of alternating high and low flow regions of approximately 50–100 μm in length of tracer labeling. This coexistence of macro- and micro-regions of flow areas was also in agreement with recent studies and suggests the possibility that localized ultrastructures within the TM and Schlemm’s canal may contribute to the overall outflow resistance around the circumference of the TM.[37,51]

To further address the question of whether charge of the fluorescent tracer may have a preferential fluid flow, due to charge-specific interactions with ECM components in the outflow pathway, tissues were sequentially perfused with amine- and carboxy-modified fluospheres. Then they were immunostained with an antibody to versican and imaged by confocal microscopy. The amount of overlapping signal between the fluospheres and the versican immunostain was quantitated in high and low flow regions. The present study used versican immunostaining to visualize ECM structures within the TM near Schlemm’s canal. Since versican is substituted with negatively charged chondroitin sulfate chains, we hypothesized that it would preferentially colocalize with the amine-modified fluorescent microspheres. Pearson’s correlation coefficients, which determine the amount of overlapping signal between two channels, were calculated between the carboxy- or the amine-modified fluospheres, and versican immunostaining in the TM. In this study, it was of interest to note that although the carboxy- and amine-modified fluospheres showed a moderate degree of colocalization with each other, both had a very weak association with versican immunostaining. Thus, carboxy- and amine-modified fluorescent microspheres localized to similar areas along the TM suggesting the existence of diverse binding sites within common fluid flow routes of the TM en route to Schlemm’s canal and the venous drainage tract.

Studies have confirmed the presence of approximately 25–30 collector channels per eye and determined them to be distributed somewhat unevenly around the circumference of Schlemm’s canal.[52–54] Preferential fluid flow has previously been reported to occur near collector channels, particularly in pigmented areas of the TM.[25,47] Recent studies corroborated this finding where high tracer-labeled regions coincided with collector channels. In this study, although we did not rigorously evaluate the relationship of tracer labeling with the presence of collector channels, we observed that the fluorescent tracer often accumulated in the JCT in regions near collector channels in both high and low flow regions. These observations are in general agreement with several previous studies showing a modest positive correlation of flow and the presence of collector channels.[25,37,47] However, based on our observations, collector channels and flow regions are not strictly correlated. In other words, if a map of collector channels were to be superimposed on the segmental flow regions of an individual eye, it is likely that there would be an equal chance of finding collector channels in the high flow as in the low flow regions. Thus, the relationship of the presence of collector channels and preferential fluid flow may be more complex than previously thought. Carefully-designed statistical regional analyses will be necessary to further clarify the relationship between collector channels and segmental outflow patterns.

Some studies have identified expression differences in ECM components in glaucomatous TM versus normal TM, such as cochlin,[55], matrix gla protein and type V collagen[56], and MMP-1, -10 and microfibril-associated glycoprotein-2[57]; however, these expression differences have not been correlated to segmental flow, except for cochlin which was found in deposits in a segmental manner. Additionally, glaucomatous TM tissue was shown to be stiffer than normal TM tissue, as measured by atomic force microscopy[58], and this difference is thought to be due to differences in ECM components. Studies are currently underway to determine whether there are stiffness differences in the segmental regions of the TM. Apart from versican and SPARC, little is known about the molecular structure and ECM components as they relate to segmental outflow.[17,29] The ECM proteins fibronectin and hyaluronan binding protein were found to have variable patterns of immunostaining, but were not correlated with tracers.[11,40] In order to better understand the ECM involvement in segmental flow, PCR arrays were used to determine ECM gene expression level differences. Several collagen genes, namely COL1A1, COL4A2, COL6A1, COL6A2, and COL16A1, were more than 1.5-fold higher in expression in high flow regions compared with low flow regions. Conversely, COL15A1 was enriched in low flow regions. Mutations in collagen genes have been associated with glaucoma (recently reviewed).[59] Of particular relevance to this study is the observation that genetic variations in COL15A1 can modify the age of onset of early and late onset primary open angle glaucoma.[60–62] Type XV collagen is a fibril-associated collagen that is a component of the basement membrane and may be involved in outflow resistance. Other ECM components such as CTNNB1, MMP2, and MMP3 genes were also more than two-fold higher in expression in high flow regions than in low flow regions. In contrast, the laminins, LAMA3, LAMB3, and LAMC1, as well as MMP16 were all enriched in low flow regions compared with high flow regions. Many previous studies have identified key ECM genes expressed in TM cells and tissues.[2,9,10,63–66] However, this study sheds new light on ECM gene-specific differences in high and low flow regions of the TM.[2,10,20,64]

High flow regions of the tissue appear to show no significant difference in total MMP proteolytic activity than low flow regions; however, the substrate used was a generic MMP substrate. Increases in activity of one MMP may be tempered by decreases in activity of another MMP. More MMP assays using specific MMP substrates may detect differences in individual MMP activities in each segmental outflow region. Previously, we had shown that, unlike many other adult tissues, the TM has high levels of MMP expression.[66–68] We recently proposed that this high MMP expression may be essential for maintenance remodeling of the outflow channels to aid passage of aqueous humor to Schlemm’s canal.[3] For instance, debris from aqueous humor may become trapped on the sticky ECM within the outflow channels as fluid passes through the tissue. MMPs may be used to cleave ECM molecules to release the debris and associated ECM fragments in order to prevent blockage and IOP elevation. The PCR array data presented here support the contention that MMPs are likely involved in outflow resistance. The use of new MMP substrates that target individual MMPs will help to dissect which enzyme activities play a role in specific regions of the tissue.

The general acknowledgement of segmental outflow should have significant future impacts on the design and delivery of treatments for glaucoma since high flow regions are inherently over-treated, while low flow areas are under-treated by current drug delivery techniques.. Thus, it will be of importance to determine molecular differences in high and low flow regions that can then be manipulated or targeted individually in future treatments of glaucoma.

## Supporting Information

S1 Fig Segmental flow is independent of tracer size and modification. Confocal images of frontal sections from perfused human anterior segments show representative high (A, C) and low (B, D) flow areas of the TM. In high flow region, Qtracker-655 (20 nm with HIV-TAT modification) (A) is selectively taken up by the outer uveal/corneoscleral TM and thus little labeling is seen in the JCT. In contrast, the 200 nm amine-modified fluorescent microspheres were evenly localized throughout the TM (C). Immunostaining for fibrillin-1 is shown in green and primarily labels the JCT region in all panels. DAPI staining is blue in all panels. Scale Bar = 50 μm. OW = Outer wall, IW = Inner wall, SC = Schlemm’s canal, JCT = Juxtacanalicular TM.
(TIF)

S2 Fig Perfusion of labeled fluorescent microspheres selectively targets the TM. Anterior segments were either sequentially perfused with 200 nm amine-modified (red) and carboxy-modified (green) fluospheres (A–C) or passively soaked with 200 nm amine-modified fluospheres for 20 hours followed by perfusion of 200nm amine-modified fluospheres (green) (D–F). Red and green channels were merged (C and F) and blue color is autofluorescence of TM. Scale Bar = 100 μm. OW = Outer wall, SC = Schlemm’s canal, IW = Inner wall.
(TIF)

S1 Table Donor eye information for all tissues used in study. All relevant biological information regarding the donor and known ocular history is listed.
(DOCX)
