# Supplementary figures and images for: Mapping Molecular Differences and Extracellular Matrix Gene Expression in Segmental Outflow Pathways of the Human Ocular Trabecular Meshwork
Source: PLoS One. 2015 Mar 31;10(3):e0122483. doi: 10.1371/journal.pone.0122483 (PMC4380331; doi:10.1371/journal.pone.0122483)

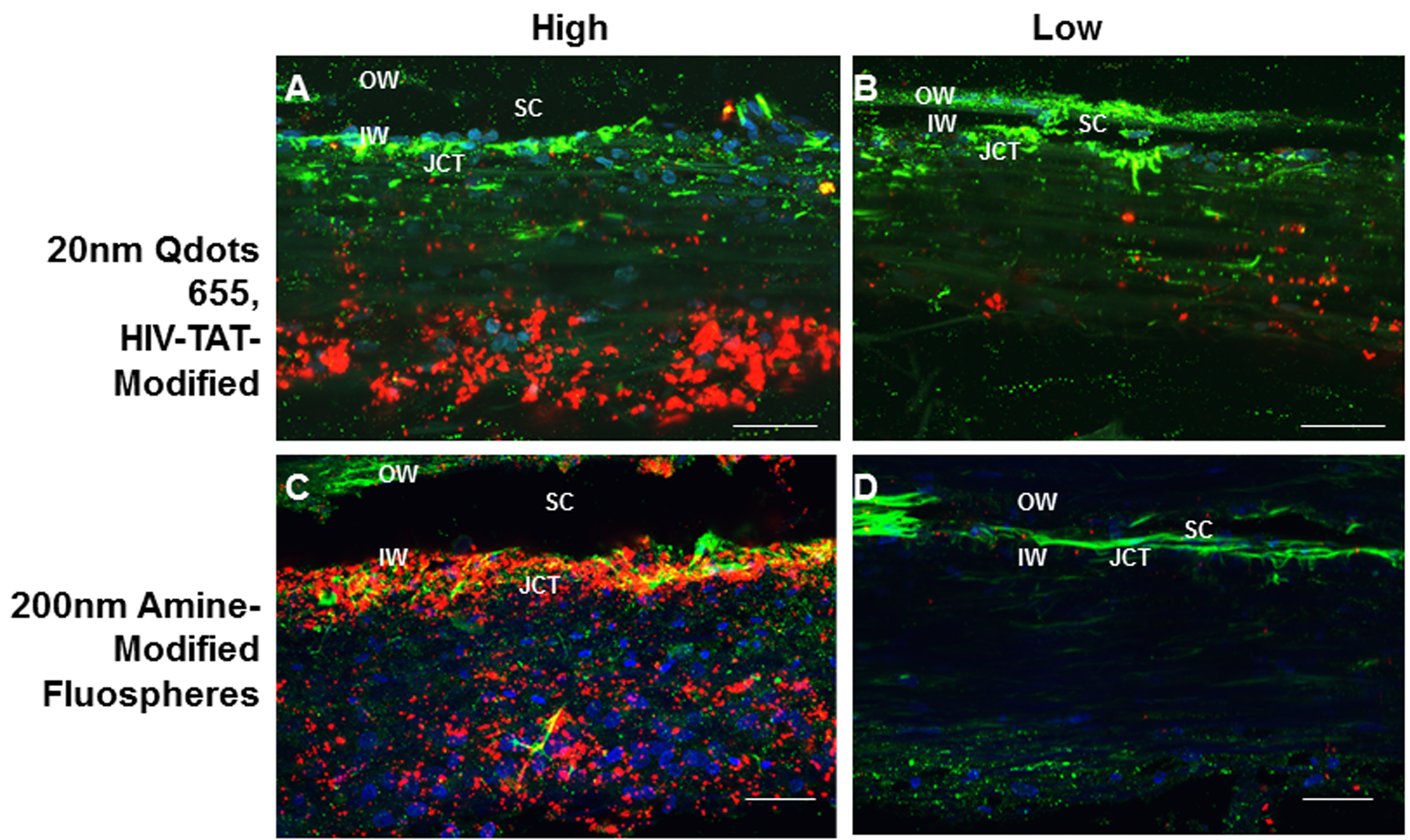

Supplement: S1 Fig — Confocal images of frontal sections from perfused human anterior segments show representative high (A, C) and low (B, D) flow areas of the TM. In high flow region, Qtracker-655 (20 nm with HIV-TAT modification) (A) is selectively taken up by the outer uveal/corneoscleral TM and thus little labeling is seen in the JCT. In contrast, the 200 nm amine-modified fluorescent microspheres were evenly localized throughout the TM (C). Immunostaining for fibrillin-1 is shown in green and primarily labels the JCT region in all panels. DAPI staining is blue in all panels. Scale Bar = 50 μm. OW = Outer wall, IW = Inner wall, SC = Schlemm’s canal, JCT = Juxtacanalicular TM. (TIF) [file pone.0122483.s001.tif]

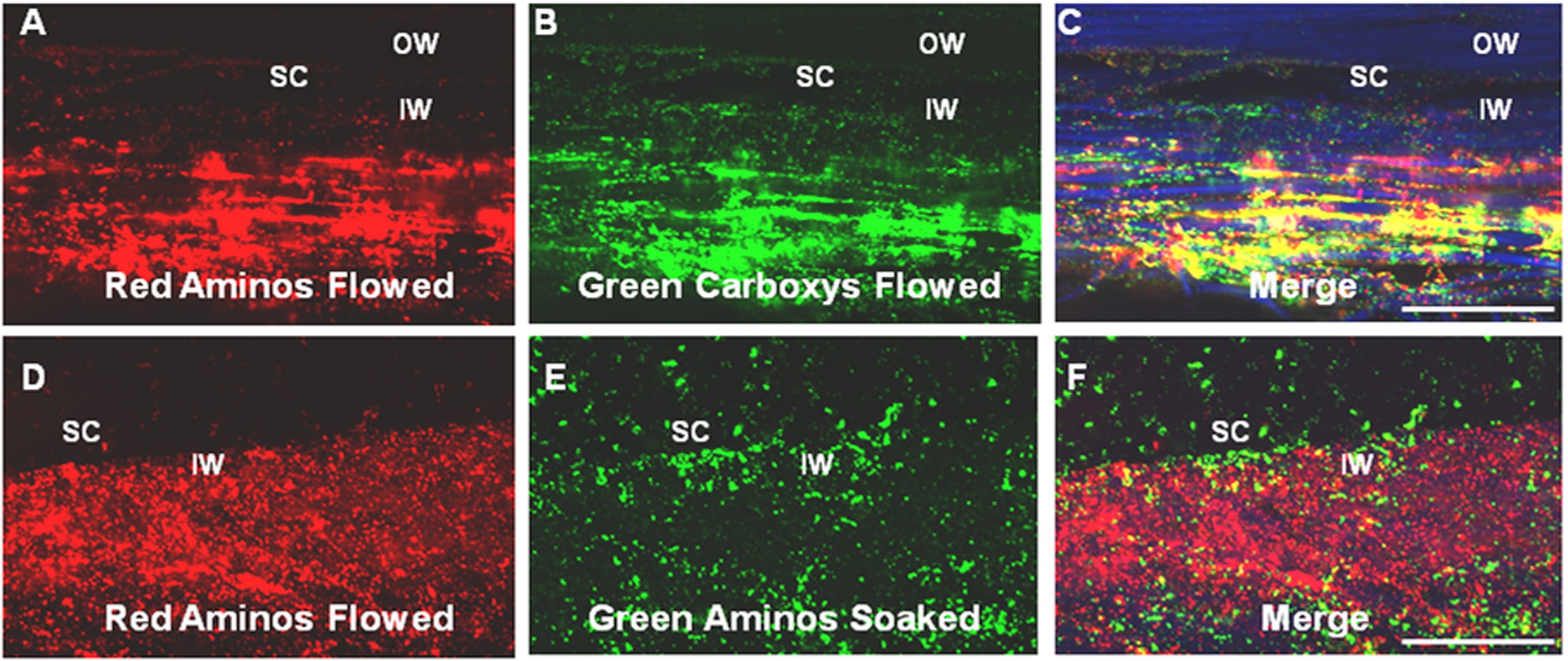

Supplement: S2 Fig — Anterior segments were either sequentially perfused with 200 nm amine-modified (red) and carboxy-modified (green) fluospheres (A–C) or passively soaked with 200 nm amine-modified fluospheres for 20 hours followed by perfusion of 200nm amine-modified fluospheres (green) (D–F). Red and green channels were merged (C and F) and blue color is autofluorescence of TM. Scale Bar = 100 μm. OW = Outer wall, SC = Schlemm’s canal, IW = Inner wall. (TIF) [file pone.0122483.s002.tif]
