# Supplementary material for: Mapping Molecular Differences and Extracellular Matrix Gene Expression in Segmental Outflow Pathways of the Human Ocular Trabecular Meshwork
Source: PLoS One. 2015 Mar 31;10(3):e0122483. doi: 10.1371/journal.pone.0122483 (PMC4380331; doi:10.1371/journal.pone.0122483)
Supplement: S1 Table — All relevant biological information regarding the donor and known ocular history is listed. (DOCX) [file pone.0122483.s003.docx]

**Table S1.** **Donor Eye Information for all tissues used in study.**

| Identifier | Age | Gender | Race | FlowRate  (µl/min) | Ocular Information |
| --- | --- | --- | --- | --- | --- |
| 2013-0081OS | 79 | Male | Caucasian | 2.45 | Ocular Normal |
| 2013-0081OD | 79 | Male | Caucasian | 2.61 | Ocular Normal |
| 2013-0086OS | 70 | Male | Caucasian | 1.38 | Ocular Normal |
| 2013-0086OD | 70 | Male | Caucasian | 2.15 | Ocular Normal |
| 2013-0087OS | 84 | Male | Caucasian | 1.04 | Ocular Normal |
| 2013-0087OD | 84 | Male | Caucasian | 8.5 | Ocular Normal |
| 2013-0276OS | 79 | Female | Caucasian | 6.43 | IOL* |
| 2013-0270OD | 97 | Female | Caucasian | 1.45 | IOL* |
| 2013-0272OS | 68 | Female | Caucasian | 1.32 | Ocular Normal |
| 2013-0272OD | 68 | Female | Caucasian | 2.68 | Ocular Normal |
| 2013-0996OS | 81 | Female | Caucasian | 6.17 | IOL* |
| 2013-0863OS | 65 | Female | Caucasian | 1.74 | IOL* |
| 2013-0417OS | 72 | Female | Caucasian | 6.43 | IOL* |
| 2013-0417OD | 72 | Female | Caucasian | 2.43 | IOL* |

* IOL = Intraocular lens surgery
